# Bibliography

**Published:** 1900-05

**Authors:** 


### Bibliography.

The Practice Builder. A Treatise on the Conduct and Enlargement of Dental Practice. By Charles R. Hambly, D.D.S., author of "The American Dental Instructor," "The British Dental Instructor," etc. Fifth Edition. Cincinnati and New York, American Dental Publication Co., 1897.

This book was prepared by the author, as he expresses it, "because it is needed."

This the fifth edition is marked on the title-page 1897. Why it has not received extended notice previously must be explained by the author. The fact that it has reached five editions indicates "that it fills a want long felt" by a certain class of operators.

It seems a formidable task for the reader to wade through six hundred and sixty-eight pages devoted to advice in building and maintaining a dental practice, and doubtless the first thought on picking up this bulky volume will be that the author has simply used a mass of verbiage to express a few ideas. This conception would, however, be very far from the truth. While it must be acknowledged that the book has been extended beyond all reasonable limits, and might be reduced to one-third its size to advantage, it contains much that is valuable, and generally so clearly stated that the reader finds himself unconsciously following in the train of the author's thought without criticism as to the redundancy of words and the repetition of ideas.

That a book of this kind is a need not only in dentistry, but in medicine, must have been apparent to all observers, and it is with regret that the author has seen fit to combine with the good much that must be condemned. Professional men are, as a rule, poor business men. A few have had a preliminary training in this direction, and they are generally successful, but the majority enter the professions direct from the schools, with the result that they fail to unite business habits with their professional work. It is not at all uncommon to find professional men incapable of opening a bank account or to keep it properly when opened.

There is no question of more importance to the young graduate than that of conducting a practice, and the voluminous advice contained in this book should aid in this, for there is much in it to which no exceptions can be made. It must be accepted as a truism that, however much a professional man may desire to separate himself from trade, the methods that insure success there must, to some extent, be adopted if satisfactory results are to be obtained.

The book opens with the Code of Ethics of the American Dental Association adopted August, 1866. This is well, but the author fails to live up to it on page 423, where he gives a chapter on "Advertising," which from the reviewer's stand-point is not only at variance with the aforesaid code, but is contrary to professional thought and practice. While it is true many men quietly violate the code, and secure, through devious ways, notices in the press, it does not, therefore, follow that the question has not an important ethical side. The author, in discussing this subject, seems to feel "that there are two kinds of advertising,—good and bad,"—and then proceeds to explain the difference, and devotes large space to informing the beginner and practitioner how to advertise intelligently. From a business point of view no exception can be made to his advice, but when he gives examples, after the manner of the "Ready Letter-Writer," how to write advertisements, he steps over the boundary of ethics into the region of "dental parlors," and thus divorces his work from professional right thinking.

This portion of his book is considered here, for in the reviewer's opinion it forms the worst feature of an otherwise generally satisfactory publication. That it will be a veritable treasure-house to the advertising dentist is assured. Pages are given to illustrate the author's opinion of what constitutes "sensible, argumentative announcements," and then follows dental parlor advertisements in America, England, and on the Continent. While the motive of the author may be to discuss good and bad methods of advertising, the true professional man will have nothing to do with the book, for to his mind the good in it is completely neutralized by this chapter. Were it not for this good in a higher business sense, this book could not have found a place on our reviewing pages.

Aside from this there is an element of mystery about it that is not understood. It is handsomely printed upon coated paper, presenting a volume unexceptionable in general make-up, and yet the author places on the cover the word "Confidential." Why this, if intended for the profession at large?

Had the author presented this book free from the objections referred to, a more favorable review would have been given, but as it stands, it must be regarded as contributing to the worst features of modern dental practice, illustrated by the dental parlors of all our large and most of the smaller cities, lowering the standard of dentistry as never before in its history.

